# Factors Associated With Depression and Anxiety in People With Rare Diseases During COVID-19: A Cross-Sectional Study

**DOI:** 10.1155/da/9002779

**Published:** 2025-05-22

**Authors:** Laura Inhestern, Alba Schwab de la O, David Zybarth, Maja Brandt, Ramona Otto, Martin Härter, Corinna Bergelt

**Affiliations:** ^1^Department of Medical Psychology, University Medical Center Hamburg, Eppendorf, Germany; ^2^German Center for Child and Adolescent Health, Partner Site Hamburg, Hamburg, Germany; ^3^Institute of Health Care Research in Dermatology and Nursing, University Medical Center Hamburg, Eppendorf, Germany; ^4^Department of Medical Psychology, University Medicine Greifswald, Greifswald, Germany; ^5^German Center for Child and Adolescent Health, Partner Site Greifswald/Rostock, Greifswald, Germany

**Keywords:** anxiety, burden, COVID-19, depression, psychological distress, psychosocial, quality of life, rare disease

## Abstract

**Background:** People living with a rare disease are a vulnerable patient group and experience challenges in participation and healthcare. Due to changes in healthcare and threat of the infection during coronavirus disease 2019 (COVID-19) pandemic, people living with rare diseases have been particularly affected. Therefore, this study aimed to investigate depressive symptoms and symptoms of anxiety during the COVID-19 pandemic and identify factors associated with symptom levels.

**Methods:** One-hundred and seventy-two people living with a rare disease were recruited from centers for rare diseases and patient organizations in Germany from January 2021 to January 2022. In addition to descriptive analyses and group comparisons, we applied multiple linear regression models to identify factors associated with outcome variables of interest (depressive and anxiety symptoms, assessed by the Hospital Anxiety and Depression Scale [HADS]).

**Results:** For the depressive symptoms, 14% of the participants reached the cutoff for moderate and 14.5% for a high level of depressive symptoms. Concerning anxiety symptoms, 22% reported moderate levels of anxiety and 13.4% reported high levels of anxiety. Higher depressive symptoms were significantly associated with older age, lower socioeconomic status, having severe or varying symptoms compared to low symptom severity, lower treatment satisfaction, lower social support, and more unmet needs. Higher anxiety levels were associated with more unmet needs and more intense COVID-19-related concerns.

**Conclusions:** The findings indicate red flags of high symptoms that should be considered during routine care of patients with rare diseases. Healthcare providers should be sensitized for the need for psychosocial support and use a quick assessment to assign patients in need to specific support programs.

**Trial Registration:** German Clinical Trials Registry: DRKS00020488

## 1. Background

Worldwide ~3%–6% of the population live with one of the over 6000–8000 different diagnoses that are categorized as rare diseases [1]. Rare diseases can vary widely with regard to symptoms; however, similar psychological burden can arise due to rarity, complexity, as well as the progressive course of disease or life-threatening condition [2]. Moreover, restricted access to treatment, care coordination, or lack of causal treatment can be burdensome to persons affected [3–5]. Due to these challenges, people living with rare diseases report an increased mental burden and reduced quality of life [6, 7]. For instance, in a European-wide survey with 3071 respondents, three times more people with a rare disease reported feeling unhappy or depressed compared to the general population [8]. In a meta-analysis, Uhlenbusch et al. [7] examined the prevalence of affective and anxiety disorders in people with rare diseases. Although prevalence rates varied widely across studies, even the lower conservative limit of the pooled estimated prevalence was shown to be higher than in the general population (15.4% vs. 9.3%). The meta-analyses identified female gender and age (older age for depression, younger age for anxiety) as risk factors for depression and anxiety [7]. A study by Bogart and Dermody [3] identified clusters based on disease characteristics (e.g., symptom onset, disease course, pain) that were associated with distress level. Similarly, a cross-sectional study by Uhlenbusch and colleagues revealed significant associations of higher symptom severity with higher symptom levels of anxiety and depression [9].

In Germany, in the early phases of the coronavirus disease 2019 (COVID-19) pandemic contact restrictions, social withdrawal, limited access to health care, and other restrictions were implemented [10–12]. Due to multiple stressors, people living with a rare disease are a vulnerable patient group who nevertheless receive little social and scientific attention due to the characteristics of their disease. The corona pandemic has had a major impact on the lives of those affected due to the difficulties in accessing care, which is highly relevant for people with rare diseases, as well as the risk of infection and associated concerns about worsening conditions and unclear effects of infection [13].

We carried out the present analyses in order to investigate psychological impact and potential influencing factors and thus investigate the consequences of the pandemic on this vulnerable patient group. As symptoms of anxiety and depression are the most prevalent symptoms and have gained particular attention during the pandemic, we focused on these outcomes. Based on the reviewed research, the following hypotheses were formulated:

Hypothesis 1: The female gender, older age, early symptom onset, severe and varying symptoms, low levels of social support, and an increased level of concern due to COVID-19 are significantly associated with high levels of depressive symptoms.

Hypothesis 2: The female gender, younger age, early symptom onset, severe and varying symptoms, low levels of social support, and an increased level of concern due to COVID-19 are significantly associated with high levels of anxiety symptoms.

## 2. Methods

This study was a secondary analysis of data from the research project “Interface Management Concepts in Healthcare for Rare Diseases in Germany” funded by the Federal Ministry of Health (Bundesministerium für Gesundheit) in Germany [14, 15]. The mixed methods study assessed experiences of both patients and healthcare providers and evaluated management concepts for intersectoral collaboration and communication.

### 2.1. Participants and Data Collection

Participants were recruited from preselected centers for rare diseases, which have been selected based on a positive pre-evaluation of their intersectoral management concepts [14, 15]. Specialized outpatient clinics within the selected centers for rare diseases invited their patients or caregivers of pediatric patients to participate in the study. In case of interest in study participation, patients and caregivers received detailed study information, and a paper–pencil questionnaire and a franked return envelope addressed to the study team. Additionally, patients were invited to participate in the study via patient organizations. Interested patients contacted the study team and received the study documents via mail. Data collection took place from January 2021 to January 2022.

For the presented analyses, only self-reported data from people with rare diseases were analyzed. Data from caregivers of pediatric patients were excluded.

### 2.2. Instruments

Depressive symptoms and anxiety symptoms were assessed using the German version of the Hospital Anxiety and Depression Scale (HADS-D), which has been developed specifically to measure symptoms of depression and anxiety in patients with somatic diseases [16, 17]. The HADS shows good psychometric properties and had been widely used [18]. The overall scale includes 14 items that refer to the presence of symptoms during the last week including the two subscales “depression” and “anxiety” with seven items each. Participants answered all items on a four-point Likert scale, leading to possible overall scores from 0 to 21 for each subscale. Recommended cutoffs categorize answers into normal (0–7), moderate (8–10), and high (11 or above) levels of anxiety and depression.

Sociodemographic and disease-related variables were assessed by patient self-report. All study participants were asked to report on the following variables: gender, age, income, education, employment, diagnosis, the two most severe symptoms and symptom severity (0 = low to 5 = highest, 6 = varying severity), and age at symptom onset. Symptom severity was dummy coded for further analyses: low severity (0–2), medium severity (3), and high severity (4–5) or high variability (6) in at least one of the two reported most severe symptoms. Socioeconomic status was measured using the Winkler–Stolzenberg Index-based on educational status, occupational status, and household income [19]. The index ranges from 3 to 21 with low status (score 3–8), middle (score 9–15), and high status (score 15–21).

To investigate treatment satisfaction, we used the ZAPA questionnaire assessing satisfaction with outpatient care [20]. It is an economic instrument, which comprises four items with a four-point Likert scale. The overall satisfaction can be calculated using the sum score of all items (possible range: 0–12). Study participants were instructed to focus on the specialized health care of their rare disease when answering the questions.

Social support was assessed using the German version of the Oslo Social Support Scale on the extent of perceived social support [21, 22]. This brief instrument (three items) has been shown to be valid and feasible [21, 23, 24].

Using the screening tool for supportive care needs, unmet needs of participants were assessed in the domains of psychological, informational, physical, support, and sexuality needs [25]. Participants rated their need for support and the extent of support needed. For our analyses, a simple scoring was used counting reported unmet needs, irrespectively of the extent of the need.

Additionally, COVID-19-related aspects were assessed by self-developed questions. Participants were asked about changes in their health care and corresponding consequences in their health status. Moreover, they were asked about their concerns regarding the pandemic about their healthcare (“Are you concerned about your health-care in the context of the COVID-19 pandemic?”, Likert scale from 1 = “No, I worry as much or as little as before” to 4 = “Yes, I am very concerned about the impact of the COVID-19 pandemic on my healthcare”).

### 2.3. Analyses

Data were analyzed using SPSS Statistics 27. We conducted descriptive analyses and analyzed group differences using chi-squared tests for categorical and *t*-tests for continuous data. Effect sizes (Cohen's *d* or *ϕ*) were calculated. To identify factors associated with outcome variables of interest (depressive symptoms, anxiety), we conducted linear multiple regression analyses (see also Supporting Information Table S1). In both models (depression and anxiety), the inclusion of predictors into the regression model was carried out equivalently to the cross-sectional study from Uhlenbusch et al. [9] using a hierarchical model and simultaneous predictor entry within the blocks. In this way, it is possible to compare whether in this study psycho-social variables can explain more variance than disease-related variables. This will have implications for clinical practice in the sense that either disease-related variables or psychosocial variables need to be emphasized in routine medical counseling to detect individuals at risk. According to the three principles from Cohen et al. [26], static variables were entered into the model first; and causal priority and relevance of the predictors were considered when deciding the order of predictor variable entry [27]. In this case, demographic and disease-related variables are mostly static and probably have a causal influence on psychosocial variables. Psychosocial variables, on the other hand, might have a causal influence on the extent of COVID-19-related concerns. Moreover, disease-related variables have a stronger practical relevance because they are always assessed in routine medical consultations. To sum up, the predictors are entered into four blocks: demographic variables, disease-related variables, psychosocial variables, and COVID-19-related concerns. Assumptions for regression analyses were verified. To prevent cumulation of Type I error, the significance of independent variables was only assessed when all predictors were included into the models. We handled missing data using pairwise deletion.

## 3. Results

### 3.1. Sample Characteristics

Demographic, disease-related, and psychosocial characteristics are presented in Tables 1 and 2. Fifty-four percent of the participants were female, and mean age was 49 years. Twenty-five percent of participants had a suspected diagnosis. Most frequent diagnoses were Marfan syndrome (27%) and amyloidosis (9%). Patients with a confirmed diagnosis reported a significantly earlier symptom onset than those with a suspected diagnosis (mean: 28 years vs. 45 years). The symptom severity and psychosocial characteristics did not differ significantly between patients with a confirmed diagnosis and a suspected diagnosis. Almost 40% of the participants described changes in their healthcare/clinical care due to COVID-19 pandemic and its consequences. Patients reported concerns rather not being concerned about the healthcare (*M* = 1.8 [SD = 0.8]).

In most of the variables, there were only a few missing values except for age and symptom onset which had more than 10% missing data.

### 3.2. Depressive and Anxiety Symptoms

In total, 28.5% of the participants reported borderline (14.5%) to clinically relevant (14%) depressive symptomatology, and 35.5% reported borderline (22%) to clinically relevant (13.4%) anxiety symptomatology.

### 3.3. Factors Associated With Depressive Symptoms

The regression analyses revealed that the sociodemographic variables explained 15% of the variation in depressive symptoms (*p*  < 0.001). Adding the disease-related variables and the psychosocial variables increased the variance explanation by 17% (*p*  < 0.001) and 19% (*p*  < 0.001). COVID-19-related concerns did not lead to a significant change in *R*^2^ (*p*=0.154). The final model explained ~52% of the variance and the model showed a high goodness-of-fit with an adjusted *R*^2^ of 0.48. In the final model, higher depressive symptoms were significantly associated with older age, lower socioeconomic status, experiencing severe or varying symptoms compared to low symptom severity, lower treatment satisfaction, lower social support, and more unmet needs (Table 3).

### 3.4. Factors Associated With Anxiety Symptoms

The regression analyses on anxiety symptoms revealed that disease-related variables accounted for 7% (*p*=0.023) of the variation, the psychosocial variables explained 24% (*p*  < 0.001) and the COVID-19-related concerns an additional 3% (*p*=0.025) of the variation. Sociodemographic variables did not significantly contribute to *R*^2^ (*p*=0.057). The variables in the final model explained 39% of the variation and the model showed a high goodness-of-fit with an adjusted *R*^2^ of 0.34. In the final model, only more unmet needs and higher COVID-19-related concerns were significantly associated with higher levels of anxiety symptoms (Table 4).

## 4. Discussion

In our sample of patients living with a rare disease, we identified almost every third participant reporting elevated levels of depression symptomatology during the second year of the COVID-19 pandemic. For anxiety symptoms, 35% reported elevated levels. Compared to pooled mean values from other studies with patients with specific rare diseases from before the pandemic, the values of our sample are in a similar range (Table 5). Depending on the disease, however, the values from other studies are significantly higher (Table 5). Results might have been different at the beginning of the pandemic, as can be seen in the studies with rare disease populations [38, 39] and in the general population [40] which were conducted between 2020 and 2021.

The findings of our regression models indicate that psychosocial variables explain most of the variance for depression symptoms. Supporting our hypothesis on depressive symptoms, we identified that older age and low levels of social support were associated with higher levels of depressive symptoms. The exploratory analysis suggests that lower socioeconomic status, experiencing severe or varying symptoms compared to low symptom severity, lower treatment satisfaction, and more unmet needs are also significantly associated with higher depressive symptoms. Social support was already identified as an associated factor by other studies in common diseases [41, 42]. Still, patients living with a rare disease may experience poor social support [43] which according to our findings can potentially exacerbate depressive symptoms. Treatment satisfaction and unmet needs might be of particular relevance in the context of rare diseases due to difficult access to specialized care, delayed diagnoses or high levels of uncertainty [44]. This finding can also be supported by a study conducted previous to the pandemic, which identified several unmet needs in patients living with a rare disease [45]. As expected and consistent with other studies [3, 9], individuals with severe and varying symptoms of their rare disease displayed more depressive symptomatology than individuals with low symptom severity. Physicians treating patients with rare diseases should pay even greater attention to psychological distress in patients with very severe or varying symptoms. Contrary to other studies [3, 7] and the hypothesis, sociodemographic and disease-related variables were not significantly associated with anxiety symptoms in our model. However, higher COVID-19-related concerns were associated with anxiety. Depending on disease and treatment, during the COVID-19 pandemic persons with rare diseases had to deal, for example, with an increased risk of infection, complex healthcare organization, or disruption in healthcare [46]. Possibly, an overall personality trait related to anxiousness such as anxiety sensitivity might be underlying [47].

The study displays some limitations that must be considered when interpreting the results. The employed sampling method was a convenience sampling through centers after a pre-evaluation of their intersectoral management concepts. Thus, the sample mostly consisted of patients who were probably well supported by the attending centers and, therefore, might have experienced a lower psychological burden than other patients without a similar support system. Also, participants were mostly from middle to high socioeconomic status. These limitations are comparable to other studies in this field [3, 6, 9]. On the other hand, the almost even distribution of men and women can be regarded as a strength of this study. A possible reason could be that the study was not labeled as a mental health survey but as a survey concerning the participants' experiences in health care. Another limitation is the use of pairwise deletion in case of missing data, which is only an unbiased procedure assuming complete random missingness [48]. A bias due to missing data in the variables age and symptom severity, which were the variables that had more than 10% missing data, cannot be ruled out. Due to the cross-sectional study design, the results do not allow for any causal interpretation. However, the aim of this study was not to draw conclusions about the causal relationships, but to identify associations that have implications for the identification of patients at risk for increased anxiety and depression symptoms.

## 5. Conclusion

The study gives an insight into the levels of depression and anxiety symptoms in patients with rare diseases during the second year of the COVID-19 pandemic. The data indicate that approximately one-third of the patients report elevated levels of depressive or anxiety symptoms. In challenging circumstances such as a pandemic, healthcare professionals should be particularly vigilant in monitoring patients who may be at an elevated risk of developing mental health problems such as anxiety or depression. Assessing the needs, the social network and the current concerns may help to identify patients in need of additional support. Further factors found by our study and supported by previous literature are symptom severity and socioeconomic status which should be considered. The COVID-19 pandemic has exacerbated social isolation during times of social restrictions and lockdowns, which might have further reduced poor social participation. Our findings indicate that some of the patients experienced changes in their healthcare, which affected daily life and healthcare for some. Hence, in times of contact restrictions or risk for infection, online consultations, online support, or telephone hotlines could be suitable measures to (a) reduce feelings of social isolation, (b) reduce COVID-19-related concerns by providing information, (c) address unmet needs, and (d) increase treatment satisfaction.

## Figures and Tables

**Table 1:** Demographic and disease characteristics among 172 rare disease patients in Germany.

Characteristic	Total		Confirmed diagnosis		Suspected diagnosis		Statistical test	
	*n*	%	*n*	%	*n*	%	Test value	*p*
Total (%)	172	100	129	75.9	41	24.1		
Gender:female (%)	93	54.1	77	59.7	15	36.6	*χ* ^2^ = 6.689	**0.012** (*ϕ* = 0.20)
Years of school education (%)							LR = 3.358 (*df* = 7)	0.931 (*ϕ* = 0.13)
Up to 10	78	46.4	56	43.5	22	55		
11 or more	86	51.2	67	51.9	17	42.5		
Other	4	2.4	3	2.4	1	2.4		
Employment status (%)							LR = 10.998 (*df* = 9)	0.370 (*ϕ* = 0.24)
Full or part-time training/retraining	89 9	51.7 5.2	70 6	54.3 4.7	17 3	41.5 7.3		
On medical leave	18	10.5	15	11.6	3	7.3		
Disability pension	12	7	11	8.6	1	2.4		
Old-age pension/early retirement	33	19.2	20	15.5	13	31.7		
Other^a^	11	6.5	7	5.5	4	9.7		
Monthly income in Euro (%)							LR = 3.899 (*df* = 6)	0.718 (*ϕ* = 0.15)
Under 1250	13	7.7	10	7.9	3	7.7		
1250–<1750	13	7.7	10	7.9	3	7.7		
1750–<2250	11	6.5	10	7.9	1	2.6		
2250–<3000	39	23.2	29	22.8	9	23.1		
3000–<4000	43	25.6	34	26.8	8	20.5		
4000–<5000	30	17.9	22	17.3	8	20.5		
≥5000	19	11.3	12	9.4	7	17.9		
Received psychotherapeutic or psychological support (%)	60	37.3	43	33.3	16	39	*χ* ^2^ = 0.535	0.564 (*ϕ* = 0.06)
Symptom severity (%)							*χ* ^2^ = 0.937	0.822 (*ϕ* = 0.08)
Low	28	17.9	20	15.5	7	17.1		
Medium	31	19.9	25	19.4	6	14.6		
High	68	43.6	48	37.2	19	46.3		
Varying	29	18.6	22	17.1	7	17.1		
Diagnosis (%)							—	—
Marfan syndrome	43	26.9	43	36.1	—	—		
Amyloidosis	14	8.8	14	11.8	—	—		
Loeys–Dietz syndrome	8	5	8	6.7	—	—		
Esophageal atresia	6	3.8	6	5	—	—		
Other	48	30	48	40.3	—	—		
Not applicable	41	25.6	—	—	41	100		
Experiences of changes due to COVID-19								
Changes in clinical care^b^	67	38.9	56	44.1	10	25.0	*χ* ^2^ = 6.397	**0.041** (*ϕ* = 0.20)
If yes, which changes							LR = 3.899 (*df* = 1)	0.109 (*ϕ* = 0.21)
Closed facilities	13	20	9	13.8	4	40		
Acute COVID-19 infection or quarantine	7	10.8	6	10.9	1	10.0	LR = 0.007 (*df* = 1)	0.931 (*ϕ* = 0.01)
Canceled appointments (clinic)	21	32.3	15	27.3	6	60.0	LR = 3.877 (*df* = 1)	0.049 (*ϕ* = 0.25)
Canceled appointments (self)	30	46.2	28	50.9	2	20.0	LR = 3.488 (*df* = 1)	0.062 (*ϕ* = 0.22)
Contact restrictions (e.g., restricted visits of staff/family to support)	8	12.3	5	9.1	3	30.0	LR = 2.764 (*df* = 1)	0.096 (*ϕ* = 0.23)
If yes: Impact on the personal situation (job, family, finances, etc.)^b^	24	37.5	18	33.4	6	60.0	LR = 2.791 (*df* = 2)	0.232 (*ϕ* = 0.22)
If yes: Impact the health status/condition^b^	39	59.1	33	58.9	6	60.0	LR = 1.002 (*df* = 2)	0.606 (*ϕ* = 0.13)

*Note:* The likelihood ratio (LR) was calculated in the case that the cross-table cells had an expected frequency of <5. Bold values indicate statistical significance at *p* < 0.05.

^a^That is, currently unemployed, housewife or husband, on parental leave, or another status.

^b^Yes, a lot or yes, some.

**Table 2:** Descriptive statistics of demographic, disease related, and psychosocial variables among 172 rare disease patients in Germany.

Characteristic	Total		Confirmed diagnosis		Suspected diagnosis		Statistical test	
	*n*	*M* (SD)	*n*	*M* (SD)	*n*	*M* (SD)	Test value	*p*
Total	172	100	129	75.9	41	24.1	—	—
Age	142	49.3 (16.6)	101	47.6 (15.8)	39	53.6 (18.4)	*t* = 1.905 (*df* = 138)	0.059 **(*d* = 0.36)**
Winkler's Social Status Index	171	13.2 (4.1)	128	13.0 (4.1)	41	13.5 (4.1)	*t* = 0.612 (*df* = 167)	0.541 **(*d* = 0.11)**
Symptom onset in years	151	32.2 (23.0)	115	28.3 (22.9)	34	45.0 (19.0)	*t* = 4.297 (*df* = 64)^d^	**<0.001** **(*d* = 0.76)**
HADS—Depression	172	5.4 (4.1)	129	5.2 (4.1)	41	5.7 (4.1)	*t* = 0.683 (*df* = 168)	0.495 **(*d* = 0.12)**
HADS—Anxiety	172	6.3 (3.9)	129	6.2 (4.0)	41	6.6 (3.7)	*t* = 0.658 (*df* = 186)	0.512 **(*d* = 0.12)**
ZAPA—Treatment satisfaction^a^	162	85.6 (18.5)	123	85.4 (18.8)	38	86.8 (17.4)	*t* = 0.430 (*df* = 159)	0.668 **(*d* = 0.08)**
SCNS-ST9—Number of unmet needs	163	3.3 (3.0)	122	3.2 (2.9)	39	3.6 (3.2)	*t* = 0.750 (*df* = 159)	0.455 **(*d* = 0.14)**
OSSS-3—social support^b^	171	10.7 (2.0)	128	10.6 (2.0)	41	11.1 (1.7)	*t* = 1.405 (*df* = 167)	0.162 **(*d* = 0.25)**
COVID-19-related concerns^c^	166	1.8 (0.9)	127	1.9 (1.0)	40	1.6 (1.0)	*t* = −1.8 (*df* = 165)	0.067 **(*d* = 0.30)**

*Note*: Range of missing values 0–30. ZAPA, Questionnaire assessing satisfaction with outpatient care with a focus on patient participation; SCNS-ST9, Screening Tool for Supportive Care Needs.

Abbreviations: HADS, Hospital Anxiety and Depression Scale; OSSS-3, Oslo Social Support Scale.

^a^Higher values indicating a higher treatment satisfaction.

^b^Higher values indicating stronger levels of social support.

^c^Assessed through the item “Are you increasingly concerned about your health care in the context of the COVID-19 pandemic?” on a Likert scale from 1 = “No, I worry as much or as little as before” to 4 = “Yes, I am very concerned about the impact of the COVID-19 pandemic on my healthcare.”

^d^Calculated for a Welch's *t*-test since the homogeneity of variance assumption was not met.

**Table 3:** Hierarchical multiple regression with depression (HADS) as the dependent variable in a sample of 171^a^ rare disease patients.

Variables	Block 1 Demographic variables			Block 2 Disease-related variables			Block 3 Psychosocial variables			Block 4 COVID-19-related variable		
	*β*	95% CI	*p*	*β*	95% CI	*p*	*β*	95% CI	*p*	*β*	95% CI	*p*
Gender^b^	−0.026	[−0.192; 0.140]	0.760	−0.006	[−0.159; 0.148]	0.939	−0.054	[−0.189; 0.080]	0.425	−0.065	[−0.199; 0.070]	0.343
Age	0.331	[0.165; 0.496]	**<0.001**	0.229	[0.074; 0.384]	**0.004**	0.227	[0.088; 0.365]	**0.002**	0.215	[0.076; 0.354]	**0.003**
Social Status Index	−0.232	[−0.398; −0.066]	**0.006**	−0.147	[−0.301; 0.006]	0.059	−0.137	[−0.270; −0.005]	**0.042**	−0.134	[−0.266; −0.002]	**0.047**
Symptom severity: medium^c^	—	—	—	0.303	[0.109; 0.497]	**0.002**	0.194	[0.024; 0.365]	**0.026**	0.194	[0.024; 0.364]	**0.026**
Symptom severity: high^c^	—	—	—	0.598	[0.384; 0.813]	**<0.001**	0.397	[0.202; 0.593]	**<0.001**	0.403	[0.208; 0.597]	**<0.001**
Varying symptoms^c^	—	—	—	0.287	[0.094; 0.481]	**0.004**	0.196	[0.026; 0.366]	**0.024**	0.201	[0.032; 0.371]	**0.020**
Treatment satisfaction (ZAPA)	—	—	—	—	—	—	−0.186	[−0.327; −0.044]	**0.011**	−0.170	[−0.313; −0.028]	**0.020**
Unmet needs (SCNS)	—	—	—	—	—	—	0.179	[0.022; 0.337]	**0.026**	0.163	[0.005; 0.322]	**0.044**
Social support (OSSS)	—	—	—	—	—	—	−0.263	[−0.404; −0.122]	**<0.001**	−0.243	[−0.386; −0.100]	**0.001**
COVID-19-related concerns	—	—	—	—	—	—	—	—	—	0.101	[−0.038; 0.240]	0.154

*R* ^2^ (Adj. *R*^2^)	0.150 (0.129)			0.322 (0.289)			0.515 (0.478)			0.523 (0.482)		
*F* (*df1; df2*)	7.298 (3; 124)			9.584 (6; 121)			13.898 (9; 118)			12.827 (10; 117)		
*p*	**<0.001**			**<0.001**			**<0.001**			**<0.001**		
Δ*R*^2^	0.150			0.172			0.192			0.008		
Δ*F* (*df1; df2*)	7.298 (3;124)			10.239 (3;121)			15.591 (3; 118)			2.063 (1;117)		
*p*	**<0.001**			**<0.001**			**<0.001**			0.154		

*Note*: Bold values indicate statistical significance at *p* < 0.05.

Abbreviations: CI, confidence interval; HADS, Hospital Anxiety and Depression Scale.

^a^Deletion of one outlier based on its standardized residual (−3.2), its studentized deleted residual (−4.1), and its leverage value of 0.29.

^b^Reference group: women.

^c^Reference group: low symptom severity.

**Table 4:** Hierarchical multiple regression with anxiety (HADS) as the dependent variable in a sample of 171^a^ rare disease patients.

Variables	Block 1 Demographic variables			Block 2 Disease-related variables			Block 3 Psychosocial variables			Block 4 COVID-19-related variable		
	*β*	95% CI	*p*	*β*	95% CI	*p*	*β*	95% CI	*p*	*β*	95% CI	*p*
Gender^b^	0.096	[−0.079; 0.271]	0.280	0.119	[−0.055; 0.293]	0.179	0.046	[−0.108; 0.200]	0.559	0.027	[−0.125; 0.179]	0.724
Age	0.166	[−0.008; 0.340]	0.062	0.100	[−0.075; 0.275]	0.262	0.056	[−0.103; 0.214]	0.488	0.035	[−0.122; 0.192]	0.659
Social Status Index	−0.164	[−0.339; 0.010]	0.065	−0.108	[−0.281; 0.066]	0.222	−0.072	[−0.224; 0.080]	0.348	−0.065	[−0.215; 0.084]	0.386
Symptom severity: medium^c^	—	—	—	0.160	[−0.060; 0.380]	0.153	0.024	[−0.171; 0.219]	0.807	0.023	[−0.168; 0.215]	0.809
Symptom severity: high^c^	—	—	—	0.371	[0.128; 0.614]	**0.003**	0.120	[−0.104; 0.343]	0.292	0.129	[−0.091; 0.349]	0.247
Varying symptoms^c^	—	—	—	0.127	[−0.092; 0.347]	0.254	−0.004	[−0.199; 0.191]	0.968	0.006	[−0.186; 0.197]	0.952
Treatment satisfaction (ZAPA)	—	—	—	—	—	—	−0.111	[−0.273; −0.052]	0.180	−0.084	[−0.245; 0.078]	0.307
Unmet needs (SCNS)	—	—	—	—	—	—	0.408	[0.228; 0.598]	**<0.001**	0.379	[0.200; 0.558]	**<0.001**
Social support (OSSS)	—	—	—	—	—	—	−0.139	[−0.300; 0.022]	0.091	−0.102	[−0.264; 0.060]	0.214
COVID-19-related concerns	—	—	—	—	—	—	—	—	—	0.180	[0.023; 0.338]	**0.025**

*R* ^2^ (Adj. *R*^2^)	0.059 (0.036)			0.129 (0.086)			0.364 (0.316)			0.391 (0.339)		
*F* (*df1; df2*)	2.577 (3; 124)			2.998 (6; 121)			7.511 (9; 118)			7.516 (10; 117)		
*p*	0.057			**0.009**			**<0.001**			**<0.001**		
Δ*R*^2^	0.059			0.071			0.235			0.027		
Δ*F* (*df1; df2*)	2.577 (3; 124)			3.278 (3;121)			14.526 (3; 118)			5.170 (1; 117)		
*p*	0.057			**0.023**			**<0.001**			**0.025**		

*Note:* Bold values indicate statistical significance at *p* < 0.05.

Abbreviations: CI, confidence interval; HADS, Hospital Anxiety and Depression Scale.

^a^Deletion of one outlier based on its standardized residual (−3.2), its studentized deleted residual (−4.1), and its leverage value of 0.29.

^b^Reference group: women.

^c^Reference group: low symptom severity.

**Table 5:** Depression and anxiety scores in different study samples of rare and common disease patients before or during COVID-19.

Study sample	HADS depression *M* (SD)	HADS anxiety *M* (SD)
The current sample of rare disease patients during COVID-19 (*n* = 172)	5.37 (4.09)	6.28 (3.92)
Rare disease patients before the COVID-19 pandemic		
Benninghoven et al. [28], Marfan syndrome (*n* = 18)	7.17 (4.69)	9.61 (5.26)
Stewart et al. [29], amyloidosis (*n* = 60)	6.1 (2.0)	5.5. (3.3)
Naik et al. [30], photodermatoses (*n* = 103)	1.9 (2.1)	4.6 (4.1)
Giusti et al. [31], MEN1 (*n* = 76),	8.68 (1.62)	8.78 (2.60)
Custers et al. [32], mitochondrial diseases (*n* = 76)	5.1 (4.1)	5.2 (2.5)
Lumry et al. [33], C1-INH-HAE (*n* = 114)	2.88 (2.93)	5.48 (3.86)
Weighted mean	4.62	5.96
Common disease patients during COVID-19		
Eckford et al. [34], cancer (*n* = 621)	6.8 (4.5)	8.2 (4.4)
Massicotte et al. [35], cancer (*n* = 36)	4.0 (2.9)	6.6 (4.0)
El Otmani et al. [36], Parkinson's disease (*n* = 50)	7.90 (-^b^)	7.98 (-^b^)
Fernandez de Las Penas et al. [37], post-COVID-19 patients^a^ (*n* = 1969)	4.7 (4.8)	4.9 (5.2)
Weighted mean	5.24	5.75

*Note*: A weighted mean was calculated according to the sample sizes of the studies.

Abbreviation: HADS, Hospital Anxiety and Depression Scale.

^a^On average 8.4 months after hospital discharge.

^b^Missing information.

## Data Availability

Data are available from the authors upon reasonable request and after consultation of the data protection manager of the University Medical Center.

## Supporting Information

Supporting Information Supporting Information Table S1. Tables on coefficients of anxiety and depression.
