# Supplementary material for: Factors Associated With Depression and Anxiety in People With Rare Diseases During COVID-19: A Cross-Sectional Study
Source: Depress Anxiety. 2025 May 22;2025:9002779. doi: 10.1155/da/9002779 (PMC12122157; doi:10.1155/da/9002779)
Supplement: Supporting Information — Table S1. Tables on coefficients of anxiety and depression. [file 9002779.f1.docx]

**Appendix A. Coefficients**

**Dependent variable: Depression**

| **Coefficients** | | | | | | | | | | | | | |
| --- | --- | --- | --- | --- | --- | --- | --- | --- | --- | --- | --- | --- | --- |
| Model | | Unstandardized coefficients | | Standardized coefficients | T | Sig. | 95,0% CI for B | | Correlations | | | Collinearity statistics | |
|  |  | B | Std error | Beta |  |  | Lower Bound | Upper Bound | Zero-order | Partial | Part | Tolerance | VIF |
| 1 | (Constant) | 4,544 | 1,588 |  | 2,862 | ,005 | 1,401 | 7,686 |  |  |  |  |  |
|  | Sex | -,211 | ,688 | -,026 | -,306 | ,760 | -1,573 | 1,152 | -,037 | -,027 | -,025 | ,972 | 1,028 |
|  | Age in years | ,082 | ,021 | ,331 | 3,951 | <,001 | ,041 | ,123 | ,312 | ,334 | ,327 | ,979 | 1,021 |
|  | SES (Winkler-Score) | -,233 | ,084 | -,232 | -2,773 | ,006 | -,400 | -,067 | -,198 | -,242 | -,230 | ,978 | 1,022 |
| 2 | (Constant) | 1,259 | 1,577 |  | ,798 | ,426 | -1,864 | 4,382 |  |  |  |  |  |
|  | Sex | -,048 | ,636 | -,006 | -,076 | ,939 | -1,307 | 1,210 | -,037 | -,007 | -,006 | ,932 | 1,073 |
|  | Age in years | ,057 | ,019 | ,229 | 2,933 | ,004 | ,019 | ,095 | ,312 | ,258 | ,220 | ,918 | 1,089 |
|  | SES (Winkler-Score) | -,148 | ,078 | -,147 | -1,904 | ,059 | -,302 | ,006 | -,198 | -,171 | -,143 | ,936 | 1,069 |
|  | Symptom severity: Medium | 3,095 | 1,001 | ,303 | 3,091 | ,002 | 1,113 | 5,078 | -,048 | ,271 | ,231 | ,583 | 1,715 |
|  | Symptom severity: High | 4,935 | ,894 | ,598 | 5,522 | <,001 | 3,166 | 6,704 | ,419 | ,449 | ,413 | ,477 | 2,095 |
|  | Varying symptoms | 3,009 | 1,025 | ,287 | 2,936 | ,004 | ,980 | 5,039 | -,051 | ,258 | ,220 | ,585 | 1,708 |
| 3 | (Constant) | 11,548 | 2,620 |  | 4,408 | <,001 | 6,360 | 16,736 |  |  |  |  |  |
|  | Sex | -,446 | ,557 | -,054 | -,801 | ,425 | -1,550 | ,657 | -,037 | -,074 | -,051 | ,891 | 1,123 |
|  | Age in years | ,056 | ,017 | ,227 | 3,237 | ,002 | ,022 | ,091 | ,312 | ,286 | ,208 | ,839 | 1,192 |
|  | SES (Winkler-Score) | -,138 | ,067 | -,137 | -2,053 | ,042 | -,271 | -,005 | -,198 | -,186 | -,132 | ,919 | 1,089 |
|  | Symptom severity: Medium | 1,985 | ,880 | ,194 | 2,257 | ,026 | ,243 | 3,727 | -,048 | ,203 | ,145 | ,555 | 1,802 |
|  | Symptom severity: High | 3,277 | ,814 | ,397 | 4,025 | <,001 | 1,665 | 4,890 | ,419 | ,347 | ,258 | ,422 | 2,369 |
|  | Varying symptoms | 2,050 | ,899 | ,196 | 2,279 | ,024 | ,269 | 3,831 | -,051 | ,205 | ,146 | ,558 | 1,791 |
|  | Treatment satisfaction (ZAPA) | -,044 | ,017 | -,186 | -2,593 | ,011 | -,078 | -,010 | -,306 | -,232 | -,166 | ,803 | 1,245 |
|  | Unmet needs (SCNS) | ,246 | ,109 | ,179 | 2,252 | ,026 | ,030 | ,463 | ,510 | ,203 | ,144 | ,648 | 1,543 |
|  | Social support (OSSS) | -,566 | ,153 | -,263 | -3,702 | <,001 | -,869 | -,263 | -,453 | -,323 | -,237 | ,813 | 1,230 |
| 4 | (Constant) | 10,145 | 2,785 |  | 3,642 | <,001 | 4,629 | 15,660 |  |  |  |  |  |
|  | Sex | -,531 | ,558 | -,065 | -,951 | ,343 | -1,635 | ,574 | -,037 | -,088 | -,061 | ,881 | 1,135 |
|  | Age in years | ,053 | ,017 | ,215 | 3,065 | ,003 | ,019 | ,088 | ,312 | ,273 | ,196 | ,828 | 1,207 |
|  | SES (Winkler-Score) | -,134 | ,067 | -,134 | -2,005 | ,047 | -,267 | -,002 | -,198 | -,182 | -,128 | ,917 | 1,090 |
|  | Symptom severity: Medium | 1,981 | ,876 | ,194 | 2,262 | ,026 | ,247 | 3,715 | -,048 | ,205 | ,144 | ,555 | 1,802 |
|  | Symptom severity: High | 3,322 | ,811 | ,403 | 4,095 | <,001 | 1,715 | 4,929 | ,419 | ,354 | ,261 | ,422 | 2,372 |
|  | Varying symptoms | 2,107 | ,896 | ,201 | 2,351 | ,020 | ,332 | 3,882 | -,051 | ,212 | ,150 | ,557 | 1,795 |
|  | Treatment satisfaction (ZAPA) | -,041 | ,017 | -,170 | -2,367 | ,020 | -,075 | -,007 | -,306 | -,214 | -,151 | ,786 | 1,273 |
|  | Unmet needs (SCNS) | ,224 | ,110 | ,163 | 2,037 | ,044 | ,006 | ,442 | ,510 | ,185 | ,130 | ,635 | 1,575 |
|  | Social support (OSSS) | -,522 | ,155 | -,243 | -3,361 | ,001 | -,830 | -,214 | -,453 | -,297 | -,215 | ,781 | 1,280 |
|  | Covid-19 related concerns | ,439 | ,306 | ,101 | 1,436 | ,154 | -,166 | 1,045 | ,323 | ,132 | ,092 | ,826 | 1,210 |
|  | | | | | | | | | | | | | |

**Dependent variable: Anxiety**

| Coefficients | | | | | | | | | | | | | |
| --- | --- | --- | --- | --- | --- | --- | --- | --- | --- | --- | --- | --- | --- |
| Model | | Unstandardized coefficients | | Standardized coefficients | T | Sig. | 95,0% CI for B | | Correlations | | | Collinearity statistics | |
|  |  | B | Std error | Beta |  |  | Lower Bound | Upper Bound | Zero-order | Partial | Part | Tolerance | VIF |
| 1 | (Constant) | 6,007 | 1,600 |  | 3,755 | <,001 | 2,841 | 9,173 |  |  |  |  |  |
|  | Sex | ,753 | ,693 | ,096 | 1,085 | ,280 | -,620 | 2,125 | ,096 | ,097 | ,095 | ,972 | 1,028 |
|  | Age in years | ,040 | ,021 | ,166 | 1,885 | ,062 | -,002 | ,081 | ,139 | ,167 | ,164 | ,979 | 1,021 |
|  | SES (Winkler-Score) | -,158 | ,085 | -,164 | -1,864 | ,065 | -,326 | ,010 | -,161 | -,165 | -,162 | ,978 | 1,022 |
| 2 | (Constant) | 4,150 | 1,711 |  | 2,425 | ,017 | ,763 | 7,538 |  |  |  |  |  |
|  | Sex | ,932 | ,690 | ,119 | 1,351 | ,179 | -,434 | 2,297 | ,096 | ,122 | ,115 | ,932 | 1,073 |
|  | Age in years | ,024 | ,021 | ,100 | 1,128 | ,262 | -,018 | ,065 | ,139 | ,102 | ,096 | ,918 | 1,089 |
|  | SES (Winkler-Score) | -,103 | ,084 | -,108 | -1,227 | ,222 | -,270 | ,063 | -,161 | -,111 | -,104 | ,936 | 1,069 |
|  | Symptom severity: Medium | 1,562 | 1,086 | ,160 | 1,438 | ,153 | -,589 | 3,713 | -,033 | ,130 | ,122 | ,583 | 1,715 |
|  | Symptom severity: High | 2,927 | ,969 | ,371 | 3,019 | ,003 | 1,008 | 4,846 | ,268 | ,265 | ,256 | ,477 | 2,095 |
|  | Varying symptoms | 1,276 | 1,112 | ,127 | 1,147 | ,254 | -,926 | 3,477 | -,055 | ,104 | ,097 | ,585 | 1,708 |
| 3 | (Constant) | 9,344 | 2,870 |  | 3,256 | ,001 | 3,660 | 15,028 |  |  |  |  |  |
|  | Sex | ,358 | ,610 | ,046 | ,586 | ,559 | -,851 | 1,567 | ,096 | ,054 | ,043 | ,891 | 1,123 |
|  | Age in years | ,013 | ,019 | ,056 | ,695 | ,488 | -,025 | ,051 | ,139 | ,064 | ,051 | ,839 | 1,192 |
|  | SES (Winkler-Score) | -,069 | ,074 | -,072 | -,942 | ,348 | -,215 | ,076 | -,161 | -,086 | -,069 | ,919 | 1,089 |
|  | Symptom severity: Medium | ,236 | ,964 | ,024 | ,245 | ,807 | -1,672 | 2,145 | -,033 | ,023 | ,018 | ,555 | 1,802 |
|  | Symptom severity: High | ,945 | ,892 | ,120 | 1,059 | ,292 | -,822 | 2,711 | ,268 | ,097 | ,078 | ,422 | 2,369 |
|  | Varying symptoms | -,040 | ,985 | -,004 | -,041 | ,968 | -1,992 | 1,911 | -,055 | -,004 | -,003 | ,558 | 1,791 |
|  | Treatment satisfaction (ZAPA) | -,025 | ,019 | -,111 | -1,349 | ,180 | -,062 | ,012 | -,293 | -,123 | -,099 | ,803 | 1,245 |
|  | Unmet needs (SCNS) | ,536 | ,120 | ,408 | 4,476 | <,001 | ,299 | ,773 | ,560 | ,381 | ,329 | ,648 | 1,543 |
|  | Social support (OSSS) | -,286 | ,168 | -,139 | -1,704 | ,091 | -,618 | ,046 | -,349 | -,155 | -,125 | ,813 | 1,230 |
| 4 | (Constant) | 6,941 | 3,012 |  | 2,304 | ,023 | ,975 | 12,906 |  |  |  |  |  |
|  | Sex | ,214 | ,603 | ,027 | ,354 | ,724 | -,981 | 1,408 | ,096 | ,033 | ,026 | ,881 | 1,135 |
|  | Age in years | ,008 | ,019 | ,035 | ,442 | ,659 | -,029 | ,046 | ,139 | ,041 | ,032 | ,828 | 1,207 |
|  | SES (Winkler-Score) | -,063 | ,072 | -,065 | -,869 | ,386 | -,206 | ,080 | -,161 | -,080 | -,063 | ,917 | 1,090 |
|  | Symptom severity: Medium | ,229 | ,947 | ,023 | ,242 | ,809 | -1,646 | 2,105 | -,033 | ,022 | ,017 | ,555 | 1,802 |
|  | Symptom severity: High | 1,021 | ,877 | ,129 | 1,164 | ,247 | -,717 | 2,759 | ,268 | ,107 | ,084 | ,422 | 2,372 |
|  | Varying symptoms | ,058 | ,969 | ,006 | ,060 | ,952 | -1,862 | 1,978 | -,055 | ,006 | ,004 | ,557 | 1,795 |
|  | Treatment satisfaction (ZAPA) | -,019 | ,019 | -,084 | -1,026 | ,307 | -,056 | ,018 | -,293 | -,094 | -,074 | ,786 | 1,273 |
|  | Unmet needs (SCNS) | ,498 | ,119 | ,379 | 4,188 | <,001 | ,263 | ,733 | ,560 | ,361 | ,302 | ,635 | 1,575 |
|  | Social support (OSSS) | -,210 | ,168 | -,102 | -1,250 | ,214 | -,543 | ,123 | -,349 | -,115 | -,090 | ,781 | 1,280 |
|  | Covid-19 related concerns | ,752 | ,331 | ,180 | 2,274 | ,025 | ,097 | 1,408 | ,374 | ,206 | ,164 | ,826 | 1,210 |
|  | | | | | | | | | | | | | |
